# Association of *MMP9* and *NOS3* Polymorphisms with Distinct Clinical Forms of Juvenile Scleroderma and Characteristics of Humoral Immunity

**DOI:** 10.3390/ijms27021109

**Published:** 2026-01-22

**Authors:** Maria Osminina, Vera Podzolkova, Maria Litvinova, Natalia Geppe, Svetlana Chebysheva, Lusine Khachatryan, Natalia Golovanova, Yulia Kostina, Oksana Lazareva-Batyreva, Angelina Polyanskaya, Olga Shpitonkova, Tatiana Subbotina, Tigran Areian, Nadezhda Podchernyaeva

**Affiliations:** 1 Department of Children’s Diseases, Sechenov First Moscow State Medical University, 119435 Moscow, Russia; osminina_m_k@staff.sechenov.ru (M.O.);; 2 Department of Medical Genetics and Postgenomic Technologies, Sechenov First Moscow State Medical University, 119435 Moscow, Russia; 3 Clinic of Children’s Diseases, Sechenov’s Center of Maternity and Childhood, 119435 Moscow, Russia; 4 N.V. Sklifosovsky Institute of Clinical Medicine, Sechenov First Moscow State Medical University, 119048 Moscow, Russia

**Keywords:** juvenile scleroderma, juvenile localized scleroderma, juvenile systemic sclerosis, single nucleotide polymorphism, *MMP1*, *MMP9*, *NOS3*, rs3918242, rs1799983, genetic association, genetic factors, genetic predisposition, autoantibodies, biomarkers of fibrosis

## Abstract

Juvenile scleroderma (JS), comprising localized (JLSd) and systemic (JSSc) forms, is a rare autoimmune disorder. This study investigated associations of polymorphisms in extracellular matrix (*MMP1*, *MMP9*) and vascular homeostasis (*NOS3*) genes with JS risk and immunological phenotypes. A case–control study involved 215JS patients (194 JLSd, 21 JSSc) and 72 controls. SNPs (*MMP1* rs1799750, *MMP9* rs3918242, *NOS3* rs1799983) were genotyped using real-time PCR followed by minisequencing and mass spectrometric analysis of reaction products. Associations with disease risk, subtypes, and immunological markers were analyzed statistically. *The MMP9* (rs3918242) CT genotype was significantly associated with JLSd (OR = 2.23, 95% CI: 1.14–4.37, *p* = 0.022), showing a trend in linear facial forms. The *NOS3* (rs1799983) GG genotype demonstrated a trend toward association with JSSc (OR = 2.61, 95% CI: 0.92–7.37, *p* = 0.065). No significant associations were found for rs1799750 *MMP1* and risk of disease development. The *MMP9* risk genotype did not correlate with scleroderma-specific autoantibodies, while the *NOS3* GG genotype was associated with lower serum levels of anti-collagen IV antibodies (*p* = 0.039). Genetic associations differ for JS subtypes: *MMP9* with JLSd and *NOS3* with JSSc. Children with CT polymorphism *MMP9* (rs3918242) and with *NOS3* (rs1799983) GG genotype were found to be genetically predisposed for the development of JS.

## 1. Introduction

Juvenile scleroderma (JS) is a rare chronic autoimmune disorder encompassing both systemic (JSSc) and localized (JLSd) forms with disease onset before the age of 16 [1]. Its pathogenesis is based on a triad of processes: vasculopathy, immune-mediated inflammation, and connective tissue fibrosis [2]. Despite distinct clinical presentations, the hypothesis of potential similarities in the key pathogenic mechanisms underlying both localized and systemic forms of the disease in adults and children has been discussed in the literature [3,4,5]. However, a unified consensus on this issue has not yet been reached.

Genetic predisposition plays a significant role in the heterogeneity of JS manifestations. Associations of certain HLA system alleles (e.g., HLA-DPB1*13:01, DRB1*04:04) with the disease development have been established, which vary across different populations and correlate with clinical subtypes and autoantibody profiles [6,7,8,9,10]. Furthermore, genome-wide and candidate gene studies have identified numerous polymorphisms in genes involved in the regulation of the immune response (e.g., *IRF5*, *STAT4*, *TNIP1*), vascular tone, and fibrosis [11,12,13,14,15,16,17]. Nevertheless, data on the contribution of specific genetic variants to the development of juvenile forms of the disease, particularly in correlation with immunological phenotypes, remain scarce and contradictory.

Among candidate genes, those encoding key regulators of extracellular matrix metabolism and vascular homeostasis are of particular interest, as these processes are central to the pathogenesis of scleroderma [12,18]. These genes include matrix metalloproteinases 1 (*MMP1*) and 9 (*MMP9*), as well as endothelial nitric oxide synthase (*NOS3*). The *MMP9* promoter polymorphism (rs3918242) influences the expression of this enzyme, which degrades type IV collagen, and may be associated with localized disturbances in tissue remodeling [19,20,21]. The functional *NOS3* polymorphism (rs1799983) has been linked to endothelial dysfunction and vasculopathy—key features of the pathogenesis, especially in systemic forms of JS [22,23,24,25]. However, a comprehensive analysis of their contribution to the susceptibility to different clinical forms of JS, as well as their association with humoral immune responses, has not been sufficiently investigated.

An important yet understudied aspect is the integration of genetic data with immunological profiles of the patients. Specifically, it remains unclear whether genetic variants influencing vascular function and matrix remodeling are associated with the production of specific autoantibodies, such as those targeting extracellular matrix collagens, which are considered potential pathogenic factors and markers in scleroderma.

The aim of this study was to perform a comprehensive analysis of associations between polymorphisms in scleroderma-related genes (*MMP1*, *MMP9*, *NOS3*) and the risk of developing various clinical forms of juvenile scleroderma. An additional objective was to assess the correlation of the identified genetic associations with autoantibody profiles and fibrosis biomarkers to deepen the understanding of the molecular mechanisms underlying the clinical heterogeneity of the disease.

## 2. Results

### 2.1. General Findings

We conducted an association analysis of single nucleotide polymorphisms (SNPs) in genes encoding key regulators of extracellular matrix and vascular tone (*MMP1* (rs1799750), *MMP9* (rs3918242), *NOS3* (rs1799983)). The study cohort comprised 287 individuals: 215 patients with various clinical forms of juvenile JS and 72 pediatric control subjects. The control group consisted of conditionally healthy children (19 boys and 54 girls) without autoimmune pathology or a significant family history of rheumatic diseases.

The clinical and demographic characteristics of the patients included in the genetic analysis are detailed in Table 1.

Preliminary analysis showed that the distribution of allele and genotype frequencies for the *NOS3* (rs1799983) and *MMP9* (rs3918242) polymorphisms in our control group (N = 72) did not differ statistically significantly from the frequencies recorded for the European population in the dbSNP database (Table 2). This confirms the representativeness of the control group for further genetic analysis.

No statistically significant associations of the *MMP1* (rs1799750) gene polymorphism with the development of JLSd or JSSc were found (Supplementary Table S1). In contrast, polymorphisms in the *MMP9* (rs3918242) and *NOS3* (rs1799983) genes showed association with the genetic predisposition to JS.

### 2.2. Association of the MMP9 (rs3918242) Polymorphism with Juvenile Localized Scleroderma (JLSd)

**Figure 1:**
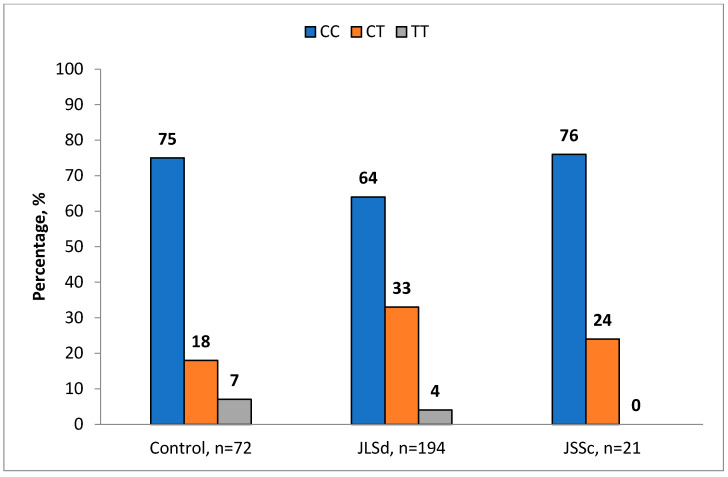
Frequency of *MMP9* (rs3918242) genotypes in JSSc, JLSd, and control groups.

A statistically significant association of the CT genotype of the *MMP9* (rs3918242) polymorphism with JLSd was identified. The frequency of this genotype in the JLSd patient group (33.0%) was significantly higher than in the control group (18.1%) (*p* = 0.022; OR = 2.23; 95% CI: 1.14–4.37) (Table 3). The allele frequencies did not differ significantly between the groups (Figure 1).

**Figure 2:**
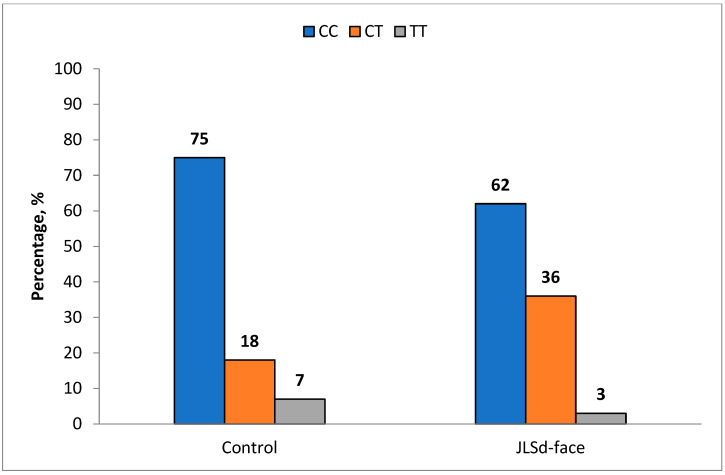
Frequency of *MMP9* rs3918242 (c.-1562C>T) genotypes in localized scleroderma patients with facial involvement (JLSd-face) and controls.

Stratification of JLSd patients by clinical forms revealed a trend toward association of the CT genotype with the linear form of scleroderma involving the face (JLSd-face). The frequency of the CT genotype in this subgroup (35.9%) was higher than in controls (18.1%), although the difference did not reach the conventional significance level (*p* = 0.059; OR = 2.54; 95% CI: 1.05–6.18)—Figure 2.

**Figure 3:**
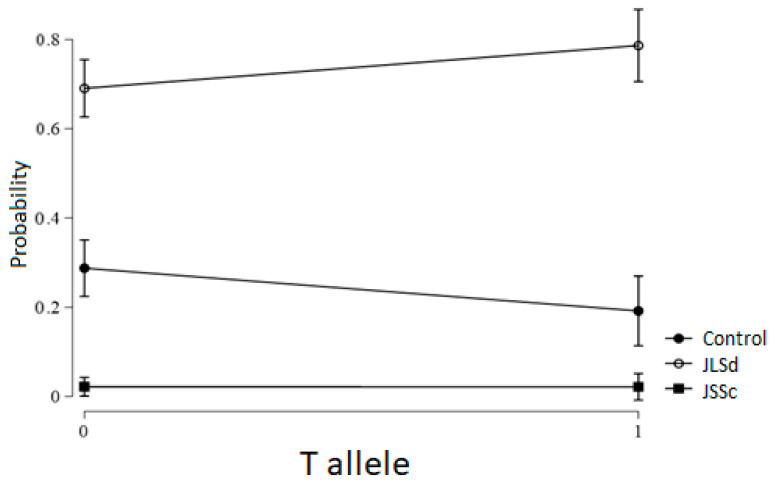
Probability of developing JSSc and JLSd in carriers of the T allele of the *MMP9* (rs3918242) gene.

Analysis of genetic inheritance models showed a trend consistent with a dominant model for the effect of the T allele on the risk of developing JLSd (*p* = 0.081; OR = 1.73; 95% CI: 0.94–3.18) (Figure 3).

Suplementary Table S3 shows the result of testing of the significance of dominant and recessive models of the influence of the T allele (rs3918242) of the *MMP9* gene on the development of JLSd.

### 2.3. Association of the NOS3 (rs1799983) Polymorphism with Juvenile Systemic Scleroderma (JSSc)

**Figure 4:**
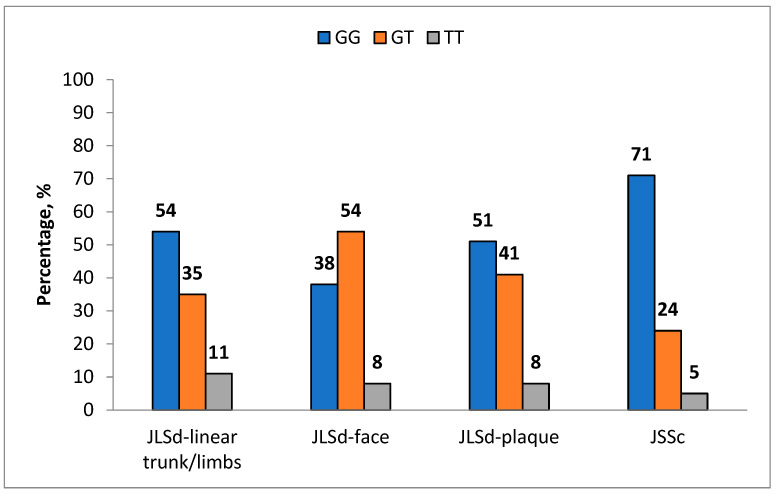
Distribution of the *NOS3* (rs1799983) SNP by disease variant.

A trend toward an association between the JS development and the GG genotype of the *NOS3* (rs1799983) polymorphism was observed in the JSSc patient group (Supplementary Table S2). The frequency of this genotype in JSSc patients was 73.9% compared to 52.1% in the control group (*p* = 0.065; OR = 2.61; 95% CI: 0.92–7.37) (Table 4, Figure 4).

The results of testing dominant and recessive genetic models for the association of the *NOS3* rs1799983 G allele with JSSc are shown in Supplementary Table S4.

### 2.4. Associations of MMP9 (rs3918242) and NOS3 (rs1799983) Polymorphisms with Patient Immunological Profiles

To assess a potential link between the identified genetic associations and features of the immune response, a comparative analysis of autoantibody profiles and fibrosis markers was performed based on *MMP9* (rs3918242) and *NOS3* (rs1799983) genotypes frequencies.

Evaluation of the *MMP9* (rs3918242) polymorphism and immunological profile revealed no statistically significant differences in the frequency of detection or mean levels of the investigated autoantibodies: RNP70, Sm, topoisomerase I, centromere B, to collagens types I-IV or in the concentrations of fibronectin and cryoglobulins when comparing patients grouped by CT genotype status (risk group for JLSd) versus CC/TT genotypes (*p* > 0.05 for all comparisons—Table 5).

Thus, while all identified CT carriers exhibited elevated fibronectin (Table 5), this represented only a non-significant trend (*p* = 0.177; Table 6). Additionally, a trend toward an association of *MMP9* (rs3918242) CT carriers with elevated levels of anti-centromere B antibodies was observed (*p* = 0.089; Table 6). Consequently, this observation requires confirmation in larger, well-defined cohorts stratified by disease subtype.

The qualitative analysis of immunological parameters and the *NOS3* (rs1799983) polymorphism showed no statistically significant associations. Only trends (*p* < 0.10) were observed for RNP70 (*p* = 0.063) and anti-collagen I (*p* = 0.066)/III (*p* = 0.083)—Table 7.

In contrast, quantitative analysis of the *NOS3* (rs1799983) polymorphism revealed a statistically significant inverse association with the level of anti-collagen IV antibodies. Carriers of the GG genotype (associated with a trend toward JSSc risk) had significantly lower concentrations of anti-collagen IV antibodies compared to carriers of GT + TT genotypes (*p* = 0.039)—Table 8.

Borderline trends toward lower levels of anti-collagen I and III antibodies were noted in the GG group (*p* = 0.055 and *p* = 0.050, respectively). No significant differences were found for other investigated laboratory parameters (*p* > 0.05).

## 3. Discussion

Our study is the first to demonstrate an association between specific genetic polymorphisms identified in peripheral blood and distinct clinical forms of juvenile JS. Furthermore, it establishes a link between these genetic variants and immunological profiles.

A key finding was the statistically significant association of the CT genotype of the *MMP9* (rs3918242) polymorphism with the predisposition to JLSd, whereas most previous studies had focused on analyzing this gene primarily in the context of JSSc [19,20]. Combined with the results of genetic model analysis, this suggests that the T allele is a risk allele for this disease form. Concurrently, we observed a trend toward an association between the GG genotype of the *NOS3* (rs1799983) gene and JSSc, which aligns with literature data on the role of this polymorphism in the development of vascular abnormalities in systemic scleroderma in adults [22,23,24,25].

The logical next step of our investigation was to analyze the potential link between the identified genetic associations and a spectrum of laboratory markers reflecting immune response activity and fibrosis. The results of this integrative analysis allow us to propose hypotheses about distinct pathogenetic pathways mediated by different genetic variants.

The absence of a statistically significant association between the CT genotype of the *MMP9* polymorphism and the profile of classic autoantibodies and fibrosis markers (except for a trend toward elevated fibronectin) is a significant result. This indirectly suggests that the association of *MMP9* (rs3918242) with the risk of developing JLSd, particularly the linear form affecting the face, may be mediated not by systemic humoral immunity but by other mechanisms. These could include local dysregulation of extracellular matrix remodeling, particularly of basement membranes [26], or disrupted neurovascular interactions in the skin. This is consistent with the known function of *MMP9* (rs3918242) as a key protease degrading type IV collagen.

In contrast to *MMP9* (rs3918242), a clear immunological correlation was discovered for the *NOS3* (rs1799983) polymorphism. Patients carrying the GG genotype (a potential risk factor for JSSc) was associated with statistically significantly lower levels of antibodies to type IV collagen and borderline significantly lower levels to types I and III collagen. This seemingly paradoxical result—lower antibody levels are associated with a more severe systemic form—finds explanation in current pathogenic models of scleroderma. The GG genotype is linked to reduced eNOS activity, pronounced endothelial dysfunction, and ischemic microvascular damage. Intensive damage to basement membranes, rich in type IV collagen, may lead to two complementary processes described in the literature: (a) rapid proteolytic degradation of the antigen with the generation of neoepitopes that may be poorly recognized in standard serological assays [27,28,29]; and (b) active formation of pathogenic immune complexes (antigen–antibody) with their subsequent deposition in tissues, which has been shown to reduce circulating free antibody levels and directly trigger endothelial damage and fibrosis [30]. Thus, a relatively low serum level of anti-collagen IV antibodies in GG carriers may reflect not a weak but an intense yet “concealed” tissue-bound humoral response directly involved in the pathogenesis of vasculopathy. This finding directly links genetically determined vascular dysfunction—via ECM damage mechanisms—with features of humoral autoimmunity in JSSc.

These findings gain particular significance in the context of existing contradictory data. While some studies in adult systemic scleroderma did not find significant associations with *MMP9* polymorphisms, our results suggest a specific role for this gene in the pathogenesis of localized disease forms, highlighting the unique genetic architecture of different JS forms [31,32,33].

The identification of *MMP9* (rs3918242) and *NOS3* (rs1799983) as potential genetic markers represents only the first step in unraveling the disease’s complex pathogenesis. A logical continuation should be the validation of these associations directly in the target tissue—the skin—where the pathological process occurs [34,35,36]. A subsequent critically important step is investigating the functional consequences of these genetic variants by analyzing their impact on the expression of corresponding genes in the affected tissue. Methodologically, the work of Khanna et al. [37] can serve as a guide; it demonstrated that therapeutic intervention with the Janus kinase inhibitor tofacitinib significantly modulates the expression profile of pro-fibrotic genes in the skin of systemic scleroderma patients.

Thus, the obtained results form the foundation for a multi-stage translational research program [4,38]. Its implementation, including tissue validation, functional analysis, and integration of genotyping data with transcriptomic profiles from serial skin biopsies during biologic therapy, will allow a shift from describing associations to a mechanistic understanding of the disease [39,40].

In summary, we present the first data on a genetic predisposition in children, linking the CT genotype of the *MMP9* (rs3918242) polymorphism and the GG genotype of *NOS3* (rs1799983) to the development of JS. The next step in our research will be to analyze a potential association between the *MMP9* (rs3918242) CT genotype and differential treatment outcomes, specifically by assessing the relative efficacy of conventional therapy (immunosuppressants and corticosteroids) versus biologic agents in these patients. This approach could open prospects for developing personalized treatment strategies, where therapy selection is guided by an individual patient’s genetic and molecular profile. This remains the ultimate goal of our research into this complex and rare pathology.

## 4. Materials and Methods

### 4.1. Study Design and Patients

This case–control study enrolled 215 patients with JS and 72 pediatric control subjects. Inclusion criteria were age under 18 years, a confirmed diagnosis of JLSd or JSSc according to current diagnostic criteria [41,42], and a minimum follow-up and evaluation period of 6 months. Exclusion criteria comprised the presence of an overlap rheumatic syndrome, mixed connective tissue disease, eosinophilic fasciitis, anogenital lichen sclerosis, and other scleroderma-like disorders.

The study was conducted in accordance with the Declaration of Helsinki and approved by the Local Ethics Committee of Sechenov University (Protocol No. 01-25, dated 23 January 2025). Informed consent was obtained from all participants and/or their legal representatives.

### 4.2. Genetic Analysis

Genomic DNA was isolated from whole venous blood samples using the DNeasy Blood & Tissue Kit (Qiagen, Hilden, Germany) on a QIAcube automated nucleic acid and protein extraction system (Qiagen, Germany) according to the manufacturer’s protocol. This study genotyped SNPs rs1799750 (*MMP1*), rs3918242 (*MMP9*), and rs1799983 (*NOS3*)—Supplementary Table S5.

Genotyping was performed by real-time polymerase chain reaction (PCR) using commercial reagent kits (Promega, Madison, WI, USA). Amplification was carried out in a 25 μL reaction mixture containing: 66 mM Tris-HCl (pH 8.8), 16.6 mM (NH_4_)_2_SO_4_, 2.5 mM MgCl_2_, 0.1 mg/mL gelatin, 250 μM of each deoxynucleotide triphosphate, 10 pmol of each primer, and 2 units of Taq polymerase (Promega, USA). The thermocycling profile was: denaturation at 94 °C for 30 s, annealing at 58 °C for 10 s, and extension at 72 °C for 20 s, for 35 cycles.

Residual dNTPs in the amplification mixture were dephosphorylated using Shrimp Alkaline Phosphatase (Fermentas, Vilnius, Lithuania). Genotyping was completed via a minisequencing (single-base extension) reaction followed by mass spectrometric analysis of the products. The minisequencing reaction was performed in a 10 μL mixture containing: 66 mM Tris-HCl (pH 9.0), 16.6 mM (NH_4_)_2_SO_4_, 2.5 mM MgCl_2_, 20 pmol of each probe, 2 nmol of a deoxy-/dideoxynucleotide mixture, and 2 units of TermiPol DNA Polymerase (Solis Biodyne, Tartu, Estonia). The reaction profile was: 94 °C for 20 s, 58 °C for 20 s, 72 °C for 15 s, for 70 cycles. Primer and probe sequences are provided in Supplementary Table S6.

Minisequencing products were purified using the SpectroCLEAN Kit (Sequenom, San Diego, CA, USA). An aliquot (0.2–1 μL) of the purified sample (10–30 pmol/μL) was applied to an AnchorChip target (400 μm, Bruker Daltonics, Bremen, Germany) pre-coated with a dried matrix. The matrix was prepared from a saturated solution of 3-hydroxypicolinic acid (Fluka, Taufkirchen, Germany) in 50% acetonitrile (Merck, Darmstadt, Germany) with the addition of 10 g/L dibasic ammonium citrate (Fluka, Germany). Mass spectra were acquired on a Reflex IV MALDI-TOF mass spectrometer (Bruker Daltonics, Germany) in linear positive ion mode using a 337 nm nitrogen laser at a 9 Hz pulse frequency. Data acquisition and primary analysis were performed with XMASS software (version 7.0, Bruker Daltonics, Germany). Subsequent spectral processing (signal-to-noise correction and precise peak mass determination) was conducted using Xtof v.1.1 (Bruker Daltonics, Germany). Alleles were determined by identifying mass spectral peaks corresponding to the expected molecular masses of the extension products. The nucleotide sequence context at the target position was established by calculating the mass differences between adjacent peaks in the spectrum.

### 4.3. Laboratory Analyses of Serum Biomarkers

Serum levels of key autoantibodies and fibrosis markers were measured. Anti-topoisomerase I (Scl-70), anti-centromere B, and anti-Sm antibodies were quantified by enzyme immunoassay using commercial kits (Organtec, Mainz, Germany). The presence of cryoglobulins was determined spectrophotometrically (PV 1251C, Solar, Minsk, Belarus) by measuring the difference in optical density at 500 nm after incubation at 4 °C and 37 °C, with values ≤ 0.06 OD considered normal. Antibodies to collagens I–IV were detected by ELISA (IMTEK, Moscow, Russia) at a 1:50 serum dilution, with positivity defined as exceeding the negative control.

Concentrations of unconjugated fibronectin and hyaluronic acid were assessed by ELISA using the Fibronectin Technoclone (Vienna, Austria) and Hyaluronic Acid Testkit Corgenix (Broomfield, CO, USA) kits, respectively, on a Multiscan FC microplate reader (Thermo Fisher, Darmstadt, Germany). Manufacturer-recommended reference ranges were applied: 70–148 mg/mL for fibronectin and ≤30 ng/mL for hyaluronic acid in children.

### 4.4. Statistical Analysis

Data were processed using Statistica 6.0 software. The normality of quantitative data distribution was assessed using the Shapiro–Wilk test. Normally distributed quantitative data are presented as mean ± standard deviation (M ± SD), while non-normally distributed data are presented as median with interquartile range (Me [Q1–Q3]). Qualitative data are presented as absolute numbers and percentages (*n*, %).

Comparisons of genotype and allele frequencies between groups were performed using Pearson’s χ^2^ test or Fisher’s exact test. Quantitative parameters were compared between two groups using Student’s *t*-test (for normal distribution) or the Mann–Whitney U test (for non-normal distribution). Differences were considered statistically significant at *p* < 0.05.

## 5. Conclusions

This study provides the first evidence of distinct genetic associations for different clinical forms of JS. We identified the *MMP9* (rs3918242) CT genotype as a potential risk factor for JLSd, implicating local dysregulation of extracellular matrix remodeling. Concurrently, the *NOS3* (rs1799983) GG genotype showed an association with the JSSc subtype, linking genetically determined endothelial dysfunction to features of tissue-bound humoral autoimmunity. These findings highlight the unique genetic architecture underlying JS subtypes and suggest divergent pathogenetic pathways.

The obtained results form a foundation for translational research. The critical next steps involve validating these genetic associations directly in the target tissue—the skin—and analyzing their functional consequences on gene expression. Furthermore, investigating the potential of these polymorphisms, particularly the *MMP9* (rs3918242) genotype, to predict differential responses to conventional versus biologic therapies represents a direct path toward personalized treatment strategies. Ultimately, this integrative approach aims to evolve from describing genetic associations to achieving a mechanistic understanding of JS, guiding more targeted and effective interventions for this complex and rare pediatric pathology.

## Figures and Tables

**Table 1:** Distribution of patients included in the genetic analysis by clinical forms of juvenile scleroderma.

Clinical Form	Total Patients	Sex (M/F) *	Subtype Details (*n*)
Juvenile Systemic Sclerosis (JSSc)	21	1/20	Diffuse cutaneous: 19 Limited cutaneous: 2
Juvenile Localized Scleroderma (JLSd)	194	52/142	Linear form, total: 84 – Trunk/limbs: 44 – Face: 40 Hemiscleroderma of trunk and extremities: 34 Plaque form, total: 68 – Circumscribed: 16 – Generalized: 52 Mixed form: 7 Pansclerotic morphea: 1
TOTAL	215	53/162	

* M—male; F—female.

**Table 2:** Comparative allele frequency of *NOS3* and *MMP9* polymorphisms in the European population and the control group.

Gene	Polymorphism (rs)	Allele	Frequency in European Population, %	Frequency in Control Group (*n* = 72), %
*NOS3*	rs1799983	G	67.9	73.5
		T	32.1	26.5
*MMP9*	rs3918242	C	83.8	90.2
		T	16.2	9.8

Data for the control group calculated from results: *NOS3* − GG = 38, GT = 31, TT = 4; *MMP9* − CC = 54, CT = 13, TT = 5.

**Table 3:** Distribution of genotypes and alleles of the *MMP9* (rs3918242) polymorphism in patients with JLSd and the control group.

Parameter	Control (*n* = 72)	JLSd (*n* = 194)	*p*-Value	OR (95% CI)
Genotype, *n* (%)			0.041 *	
CC	54 (75.0)	123 (63.4)	0.081	0.58 (0.31–1.06)
CT	13 (18.1)	64 (33.0)	0.022 *	2.23 (1.14–4.37)
TT	5 (6.9)	7 (3.6)	0.316	0.50 (0.14–1.53)
Allele, *n* (%)			0.280	
C	121 (84.0)	333 (80.4)		0.78 (0.47–1.30)
T	23 (16.0)	81 (19.6)		1.32 (0.79–2.21)

Note: * *p* < 0.05; Pearson’s χ^2^ test for genotypes (3 × 2): χ^2^ = 6.40, df = 2, *p* = 0.041.

**Table 4:** Distribution of genotypes and alleles of the *NOS3* (rs1799983) polymorphism in patients with JSSc and the control group.

Parameter	Control (*n* = 72)	JSSc (*n* = 21)	*p*-Value	OR (95% CI)
Genotype, *n* (%)			0.173	
GG	38 (52.1)	17 (73.9)	0.065	2.61 (0.92–7.37)
GT	30 (41.6)	5 (21.7)	0.173	0.38 (0.13–1.12)
TT	4 (6.3)	1 (4.3)	0.831	0.78 (0.08–7.39)
Allele, *n* (%)			0.111	
G	107 (73.3)	39 (84.8)		2.03 (0.84–4.92)
T	39 (26.7)	7 (15.2)		0.49 (0.20–1.19)

Note: Pearson’s χ^2^ test for genotypes (3 × 2): χ^2^ = 3.51, df = 2, *p* = 0.173.

**Table 5:** Frequencies and proportions (%) of autoantibody, fibronectin, and cryoglobulin indicators by *MMP9* (rs3918242) polymorphism groups.

Parameter	Category	CC + TT *n* (%)	CT *n* (%)	Total	χ^2^	*p*-Value
Anti-RNP70	Negative	44 (71.0)	18 (29.0)	62	0.026	0.873
	Positive	2 (66.7)	1 (33.3)	3		
Anti-Sm	Negative	45 (75.0)	15 (25.0)	60	0.105	0.746
	Positive	2 (66.7)	1 (33.3)	3		
Anti-topoisomerase I	Normal	68 (72.3)	26 (27.7)	94	0.025	0.875
	Elevated	7 (70.0)	3 (30.0)	10		
Anti-centromere B	Negative	71 (74.7)	24 (25.3)	95	1.213	0.271
	Positive	2 (50.0)	2 (50.0)	4		
Anti-collagen I	Normal	7 (77.8)	2 (22.2)	9	0.182	0.670
	Elevated	29 (70.7)	12 (29.3)	41		
Anti-collagen II	Normal	13 (68.4)	6 (31.6)	19	0.195	0.659
	Elevated	23 (74.2)	8 (25.8)	31		
Anti-collagen III	Normal	14 (70.0)	6 (30.0)	20	0.066	0.797
	Elevated	22 (73.3)	8 (26.7)	30		
Anti-collagen IV	Normal	27 (73.0)	10 (27.0)	37	0.067	0.796
	Elevated	9 (69.2)	4 (30.8)	13		
Fibronectin	Normal	5 (100.0)	0 (0.0)	5	2.190	0.139
	Elevated	26 (68.4)	12 (31.6)	38		
Cryoglobulins	Normal	28 (68.3)	13 (31.7)	41	0.229	0.633
	Elevated	35 (72.9)	13 (27.1)	48		

**Table 6:** Comparison of mean antibody levels, fibronectin, and cryoglobulins for different *MMP9* (rs3918242) genetic groups.

Parameter	Group	*n*	Mean ± SD	95% CI	t	*p*-Value
Anti-RNP70	CC + TT	47	6.59 ± 29.20	[−1.98–15.17]	−0.996	0.323
	CT	17	16.62 ± 49.46	[−8.82–42.05]		
Anti-Sm	CC + TT	47	7.09 ± 29.52	[−1.58–15.76]	−0.690	0.493
	CT	16	14.19 ± 49.56	[−12.22–40.60]		
Anti-topoisomerase I	CC + TT	73	9.85 ± 39.66	[0.60–19.11]	0.053	0.958
	CT	26	9.38 ± 38.89	[−6.33–25.09]		
Anti-centromere B	CC + TT	73	0.48 ± 0.48	[0.37–0.59]	−1.72	0.089
	CT	25	0.73 ± 0.98	[0.33–1.14]		
Anti-collagen I	CC + TT	36	0.39 ± 0.12	[0.35–0.43]	−0.69	0.492
	CT	14	0.42 ± 0.07	[0.38–0.45]		
Anti-collagen II	CC + TT	36	0.52 ± 0.17	[0.47–0.58]	0.12	0.906
	CT	14	0.52 ± 0.16	[0.42–0.61]		
Anti-collagen III	CC + TT	36	0.39 ± 0.12	[0.35–0.43]	0.88	0.383
	CT	14	0.36 ± 0.08	[0.31–0.40]		
Anti-collagen IV	CC + TT	36	0.28 ± 0.09	[0.25–0.31]	−0.87	0.387
	CT	14	0.31 ± 0.07	[0.26–0.35]		
Fibronectin	CC + TT	47	133.19 ± 76.58	[110.71–155.68]	−1.37	0.177
	CT	19	158.53 ± 39.71	[139.38–177.67]		
Cryoglobulins	CC + TT	63	0.062 ± 0.064	[0.046–0.078]	0.68	0.501
	CT	26	0.053 ± 0.041	[0.036–0.069]		

**Table 7:** Categorical autoantibody and biomarker profiles by *NOS3* (rs1799983) genotype (GT + TT vs. GG).

Parameter	Category	GT + TT *n* (%)	GG *n* (%)	Total	χ^2^	*p*-Value
Anti-nuclear RNP	Negative	28 (45.2)	34 (54.8)	62		
	Positive	3 (100.0)	0 (0.0)	3	3.45	0.063
Anti-Sm	Negative	29 (48.3)	31 (51.7)	60		
	Positive	2 (66.7)	1 (33.3)	3	0.38	0.535
Anti-topoisomerase I	Normal	44 (46.8)	50 (53.2)	94		
	Elevated	6 (60.0)	4 (40.0)	10	0.63	0.427
Anti-centromere B	Negative	47 (49.5)	48 (50.5)	95		
	Positive	2 (50.0)	2 (50.0)	4	0.00	0.984
Anti-collagen I	Normal	2 (22.2)	7 (77.8)	9		
	Elevated	23 (56.1)	18 (43.9)	41	3.39	0.066
Anti-collagen II	Normal	9 (47.4)	10 (52.6)	19		
	Elevated	16 (51.6)	15 (48.4)	31	0.08	0.771
Anti-collagen III	Normal	7 (35.0)	13 (65.0)	20		
	Elevated	18 (60.0)	12 (40.0)	30	3.00	0.083
Anti-collagen IV	Normal	17 (46.0)	20 (54.0)	37		
	Elevated	8 (61.5)	5 (38.5)	13	0.94	0.333
Fibronectin	Normal	3 (60.0)	2 (40.0)	5		
	Elevated	17 (44.7)	21 (55.3)	38	0.41	0.520
Cryoglobulins	Normal	21 (51.2)	20 (48.8)	41		
	Elevated	22 (45.8)	26 (54.2)	48	0.26	0.612

**Table 8:** Comparison of mean antibody levels, fibronectin, and cryoglobulins for different *NOS3* genetic groups.

Parameter	Group	*n*	Mean ± SD	95% CI	t	*p*-Value
Anti-RNP70	GT + TT	31	10.81 ± 36.95	[−2.74–24.36]	0.34	0.738
	GG	33	7.80 ± 34.70	[−4.51–20.10]		
Anti-Sm	GT + TT	31	10.03 ± 36.19	[−3.24–23.31]	0.25	0.804
	GG	32	7.79 ± 35.08	[−4.86–20.44]		
Anti-topoisomerase I	GT + TT	49	14.03 ± 48.01	[0.24–27.82]	1.08	0.283
	GG	50	5.51 ± 28.08	[−2.47–13.49]		
Anti-centromere	GT + TT	49	0.574 ± 0.714	[0.369–0.779]	0.45	0.653
	GG	49	0.515 ± 0.575	[0.349–0.680]		
Anti-collagen I	GT + TT	25	0.428 ± 0.122	[0.378–0.479]	1.96	0.055
	GG	25	0.369 ± 0.091	[0.331–0.406]		
Anti-collagen II	GT + TT	25	0.550 ± 0.200	[0.467–0.632]	1.21	0.233
	GG	25	0.493 ± 0.120	[0.444–0.543]		
Anti-collagen III	GT + TT	25	0.410 ± 0.125	[0.358–0.461]	2.01	0.050
	GG	25	0.350 ± 0.079	[0.318–0.383]		
Anti-collagen IV	GT + TT	25	0.313 ± 0.092	[0.275–0.351]	2.13	0.039
	GG	25	0.265 ± 0.066	[0.237–0.292]		
Fibronectin	GT + TT	32	141.19 ± 50.93	[122.83–159.55]	0.08	0.937
	GG	34	139.82 ± 82.84	[110.92–168.73]		
Cryoglobulins	GT + TT	43	0.056 ± 0.047	[0.041–0.070]	−0.54	0.591
	GG	46	0.062 ± 0.068	[0.042–0.082]		

## Data Availability

The data presented in this study are available upon request from the corresponding author due to being part of an ongoing study.

## Supplementary Materials

The following supporting information can be downloaded at: https://www.mdpi.com/article/10.3390/ijms27021109/s1.
