# Supplementary material for: Association of MMP9 and NOS3 Polymorphisms with Distinct Clinical Forms of Juvenile Scleroderma and Characteristics of Humoral Immunity
Source: Int J Mol Sci. 2026 Jan 22;27(2):1109. doi: 10.3390/ijms27021109 (PMC12841920; doi:10.3390/ijms27021109)
Supplement: Supplementary file 1 [file ijms-27-01109-s001.zip › ijms-4097599-supplementary.pdf]

Supplementary Table S1. Distribution of *MMP1* rs1799750 genotypes, alleles, and genetic models across Juvenile Scleroderma subtypes.

**Category / Group**	**JLSd-plaque**	**JLSd-Lin**	**JLSd-G**	**JSSc**	**Total**	**p**
GENOTYPE, n (%)	N=81	N=39	N=74	N=21	N=215	0.783
GG	23 (28.4%)	9 (23.1%)	27 (36.5%)	5 (23.8%)	64 (29.8%)	
GT	18 (22.2%)	11 (28.2%)	15 (20.3%)	5 (23.8%)	49 (22.8%)	
TT	40 (49.4%)	19 (48.7%)	32 (43.2%)	11 (52.4%)	102 (47.4%)	
ALLELE, n (%)	N=162	N=78	N=148	N=42	N=430	0.087
G	64 (39.5%)	29 (37.2%)	69 (46.6%)	15 (35.7%)	177 (41.2%)	
T	98 (60.5%)	49 (62.8%)	79 (53.4%)	27 (64.3%)	253 (58.8%)	
DOMINANT MODEL (GG vs. GT+TT), n (%)	N=81	N=39	N=74	N=21	N=215	0.413
GG	23 (28.4%)	9 (23.1%)	27 (36.5%)	5 (23.8%)	64 (29.8%)	
GT+TT	58 (71.6%)	30 (76.9%)	47 (63.5%)	16 (76.2%)	151 (70.2%)	
RECESSIVE MODEL (GG+GT vs. TT), n (%)	N=81	N=39	N=74	N=21	N=215	0.831
GG+GT	41 (50.6%)	20 (51.3%)	42 (56.8%)	10 (47.6%)	113 (52.6%)	
TT	40 (49.4%)	19 (48.7%)	32 (43.2%)	11 (52.4%)	102 (47.4%)	

* JLSd-plaque – Plaque form of Juvenile Localized Scleroderma, JLSd-Lin – Linear form of Juvenile Localized Scleroderma, JLSd-G – Generalized form of Juvenile Localized Scleroderma, JSSc – Juvenile Systemic Sclerosis.

Supplementary Table S2. Distribution of *NOS3* genotypes and alleles in the control group and patient groups with JLSd and JSSc.

**NOS3 Genotype**	**Control Group**	**Total**	**JSSc Group**	**Total**	**JLSd Group**	**Total**
GT+TT	35 (47.9%)	138 (47.9%)	5 (25.0%)	40 (43.0%)	98 (50.0%)	133 (49.4%)
GG	38 (52.1%)	150 (52.1%)	15 (75.0%)	53 (56.9%)	98 (50.0%)	136 (50.6%)
Total	73 (100%)	288 (100%)	20 (100%)	93 (100%)	196 (100%)	269 (100%)
p-value			0.065		0.764	

Supplementary Table S3. Testing the significance of dominant and recessive models of the influence of the T allele (rs3918242) of the MMP9 gene on the development of JLSd.

**Model**	**Control group (N=72)**	**Patients with JLSd (N=194)**	**p**	**OR (95% CI)**
Dominant: CC vs CT+TT	18/54	71/123	0.081	1.73 (0.94–3.18)
Recessive: TT vs CC+ CT	5/67	7/187	0.316	1.99 (0.61 – 6.50)

Supplementary Table S4. Analysis of dominant and recessive genetic models for the association of the *NOS3* rs1799983 G allele with juvenile systemic scleroderma (JSSc).

Genetic Model	Genotype Group	Control Group (N=72) n (%)	JSSc Patients (N=23) n (%)	p-value	OR (95% CI)
**Dominant**	**GG+GT vs. TT**	22 (30.6) / 1 (1.4)	69 (95.8) / 4 (5.6)	0.831	0.19 (0.05–0.77)
**Recessive**	**GG vs. GT+TT**	17 (23.6) / 6 (8.3)	38 (52.8) / 35 (48.6)	0.065	2.30 (0.80–6.59)

Note: Data are presented as counts (frequencies, %) for the genotype groups compared in each model. OR, odds ratio; CI, confidence interval.

Supplementary Table S5. Characteristics of the studied single nucleotide polymorphisms.

Gene	Polymorphism (coding DNA)	Polymorphism (protein)	rs ID
MMP1	c.-1607G>GG	–	rs1799750
MMP9	c.-1562C>T	–	rs3918242
NOS3	c.894T>G	p.Asp298Glu	rs1799983

Supplementary Table S6. Oligonucleotide primer and probe sequences for genotyping.

Gene	Primer/Probe ID	Sequence (5'→3')
MMP1	Forward primer (MMP1F1)	CTTTGGTCTCTGCCGCAC
	Reverse primer (MMP1R1)	CACCTTTCCCACTGTATCAGGT
	Probe (MMP1e+)	TGTAGTTAAATAATTAGAAA
MMP9	Forward primer (MMP9F2)	GCCTGGCACATAGTAGGCCC
	Reverse primer (MMP9R2)	CTTCCTAGCCAGCCGGCATC
	Probe (MMP9Em)	CGAGTAGCTGGTATTATAGGC
NOS3	Forward primer (n64f1)	CCACAGCTCTGCATTCAGCA
	Reverse primer (n64r1)	ATTTAGGAGGCAACCCTGGAC
	Probe (n64ep)	TGCAGGCCCCAGATGA
